# The complete chloroplast genome of the invasive and Cd-hyperaccumulator herb *Bidens pilosa* L. (Asteraceae)

**DOI:** 10.1080/23802359.2019.1704188

**Published:** 2020-01-08

**Authors:** Yu-xiang Lin, Ren-yan Duan, Zhi-xiong Tan, Yin-hua Ma, Hao Wu

**Affiliations:** College of Agriculture and Biotechnology, Hunan University of Humanities, Science and Technology, Loudi, PR China

**Keywords:** *Bidens pilosa*, chloroplast genome, Cd-hyperaccumulator, phylogenetic analysis

## Abstract

*Bidens pilosa* is an annual invasive and Cd-hyperaccumulator herb. The complete chloroplast genome sequence of the *B. pilosa* is 150,542 bp in length, which is composed of a large single-copy region of 83,542 bp, a small single-copy region of 17,624 bp and a pair of inverted repeat regions of 24,688 bp. It encodes a set of 114 genes, consisting of 80 protein coding, 30 tRNA and 4 rRNA genes. Among all of these genes, 2 genes possess double introns, and 16 genes have a single intron. *P*hylogenetic analysis showed that *B. pilosa* clustered together with *Marshallia obovata.*

*Bidens pilosa* L. (Asteraceae) is an annual invasive and Cd-hyperaccumulator herb. It is originated from tropical America (Reddy and Singh 1992). At present, it is distributed in the tropics and subtropics of Asia and America. This non-native plant is introduced into China in 1857, and now it is widely distributed in China. This invasive species can produce a large population within one to two generations after spreading to a new habitat for its high seed germination rate, efficient reproductive ability and strong phenotypic plasticity to light, temperature and nitrogen (Chauhan et al. 2019). This invasive species can directly or indirectly damage the survival of local species, agricultural production and biodiversity through interspecific competition and allelopathy (Zhang et al. 2019). *B. pilosa* can been used as the staple food or an ingredient in food for animal or human consumption (Bartolome et al. 2013) and the components of medicinal herbs to treat more than 40 diseases in humans and animals (Hsu et al. 2009; Lai et al. 2015). *B. pilosa* is a cadmium super enrichment plant and an arsenic exclusion species (Sun et al. 2009; Dai et al. 2017). It also has a strong tolerance to the combined pollution of arsenic and cadmium (Sun et al. 2009).

Fresh leaves of *B. pilosa* were obtained from the Xunyangba Town, Ningshan County, Ankang City, Shaanxi Province of China (108°32′22″E, 33°32′38″N) with voucher specimen deposited at the Herbarium of Hunan University of Humanities, Science and Technology (RW2019100501). After total genomic DNA extraction, high-throughput DNA sequencing (pair-end 150 bp) was conducted on an Illumina Hiseq X Ten platform and the sequence data were used for the assembly of cp genome with MITObim v1.9 (Hahn et al. 2013). The cp genome of *Eclipta prostrata* (KU 361242) (Park et al. 2016) was included as the initial reference.

The complete chloroplast genome of *B. pilosa* (MN 729611) is 150,542 bp in length, containing a large single copy region of 83,542 bp, a small single copy region of 17,624 bp and a pair of inverted repeat regions of 24,688 bp. The overall AT-content of the whole plastome is 62.50%. A total of 114 genes are predicted in the genome, including 80 protein-coding genes, 30 tRNA genes, and 4 rRNA genes. Among all of these genes, 2 genes (*clpP* and *ycf3*) possess double introns, and 16 genes (*atpF*, *ndhA*, *ndhB*, *petB*, *petD*, *rpl2*, *rpl16*, *rpoC1*, *rps12*, *rps16*, *trnA-UGC*, *trnG-UCC*, *trnI-GAU*, *trnK-UUU*, *trnL-UAA* and *trnV-UAC*) have a single intron.

To further investigate the phylogenetic position of *B. pilosa*, a neighbor-joining (NJ) analysis was constructed based on the concatenated chloroplast protein-coding sequences of 47 other Asteraceae species using MEGA7 (Kumar et al. 2016) with 1000 bootstrap replicates. The phylogenetic tree showed that *B. pilosa* clustered together with *Marshallia obovata* (Figure 1). This study identified the whole chloroplast genome sequence of *B. pilosa*, which may provide valuable resources for the genetic research and important guidance for the scientific management and efficient utilization of *B. pilosa*.

**Figure 1.**
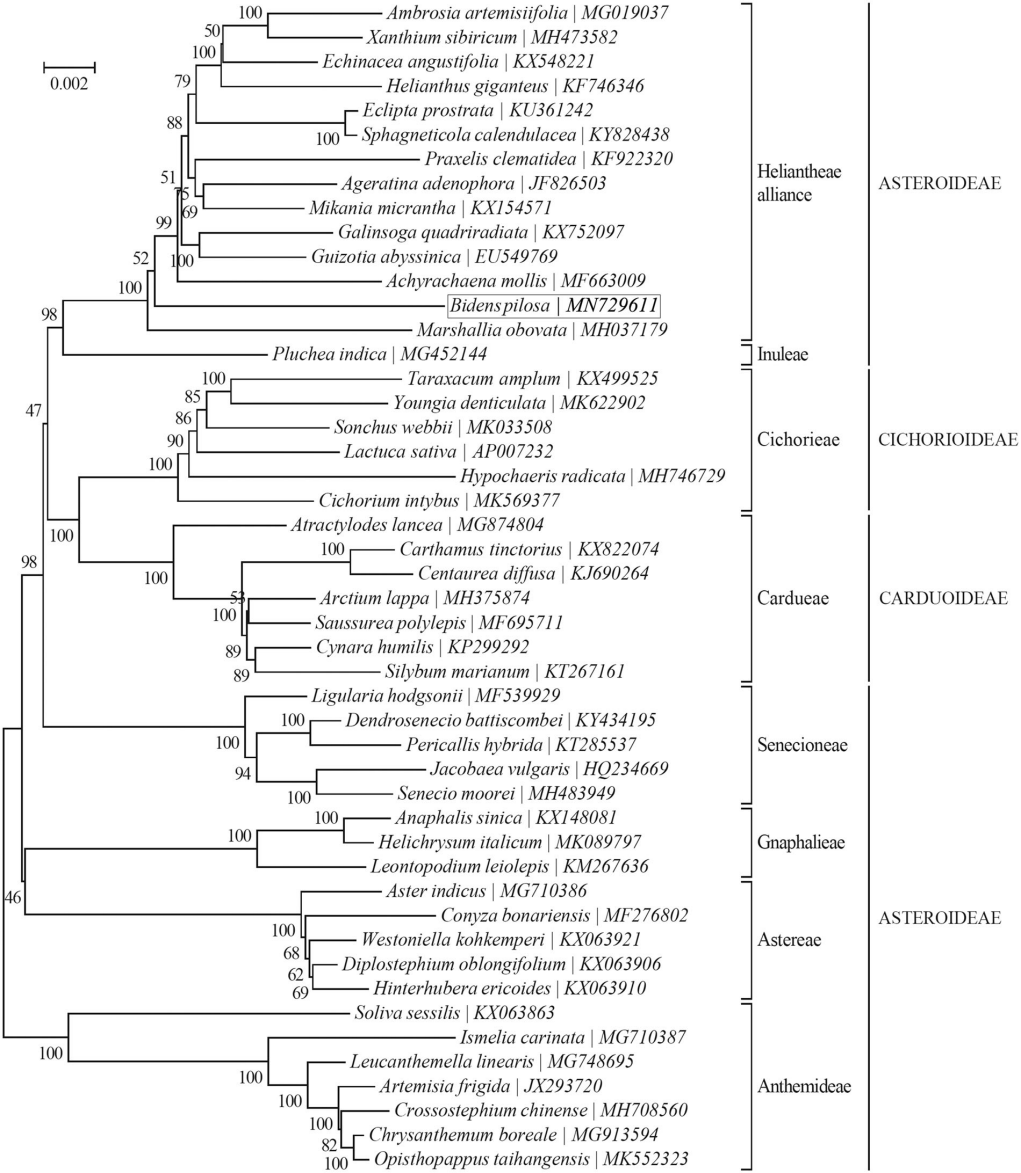
Phylogeny of 48 species within the family Asteraceae based on the neighbor-joining (NJ) analysis of the concatenated chloroplast protein-coding sequences. The support values are based on 1000 random replicates, and are placed next to the branches. The tribe- and subfamily-level taxonomy is also shown.

## References

[CIT0001] Bartolome AP, Villasenor IM, Yang WC. 2013. *Bidens pilosa* L. (Asteraceae): otanical properties, traditional uses, phytochemistry, and pharmacology. Evid-Based Compl Alt. 2013:340215. 10.1155/2013/340215 PMC3712223 23935661

[CIT0002] Chauhan BS, Ali HH, Florentine S. 2019. Seed germination ecology of *Bidens pilosa* and its implications for weed management. Sci Rep. 9(1):9. 31690889 10.1038/s41598-019-52620-9 PMC6831588

[CIT0003] Dai H, Wei S, Twardowska I, Han R, Xu L. 2017. Hyperaccumulating potential of *Bidens pilosa* L. for Cd and elucidation of its translocation behavior based on cell membrane permeability. Environ Sci Pollut Res. 24(29):23161–23167. 10.1007/s11356-017-9962-9 28828736

[CIT0004] Hahn C, Bachmann L, Chevreux B. 2013. Reconstructing mitochondrial genomes directly from genomic next-generation sequencing reads-a baiting and iterative mapping approach. Nucl Acids Res. 41(13):e129. 23661685 10.1093/nar/gkt371 PMC3711436

[CIT0005] Hsu YJ, Lee TH, Chang CL, Huang YT, Yang WC. 2009. Anti-hyperglycemic effects and mechanism of *Bidens pilosa* water extract. J Ethnopharmacol. 122(2):379e383. 19162158 10.1016/j.jep.2008.12.027

[CIT0006] Kumar S, Stecher G, Tamura K. 2016. MEGA7: Molecular Evolutionary Genetics Analysis Version 7.0 for bigger datasets. Mol Biol Evol. 33(7):1870–1874. 27004904 10.1093/molbev/msw054 PMC8210823

[CIT0007] Lai BY, Chen TY, Huang SH, Kuo TF, Chang TH, Chiang CK, Yang MT, Chang C. 2015. *Bidens pilosa* formulation improves blood homeostasis and beta-cell function in men: a pilot study. Evid-Based Compl Alt. 2015, 832314. 10.1155/2015/832314 PMC4381681 25866541

[CIT0008] Park JY, Lee YS, Kim JK, Lee HO, Park HS, Lee SC, Kang JH, Lee TJ, Sung SH, Yang TJ. 2016. The complete chloroplast genome of *Eclipta prostrata* L. (Asteraceae). Mitochondr DNA Part B. 1(1):414–415. 10.1080/23802359.2016.1176882 PMC7799467 33473502

[CIT0009] Reddy KN, Singh M. 1992. Germination and emergence of hairy beggarticks (*Bidens pilosa*). Weed Sci. 40(2):195–199.

[CIT0010] Sun YB, Zhou QX, Liu WT, An J, Xu ZQ, Wang L. 2009. Joint effects of arsenic and cadmium on plant growth and metal bioaccumulation: a potential Cd-hyperaccumulator and As-excluder *Bidens pilosa* L. J Hazard Mater. 165(1–3):1023–1028. 19070954 10.1016/j.jhazmat.2008.10.097

[CIT0011] Zhang KM, Shen Y, Yang J, Miu X, Bhowmik PC, Zhou X, Fang YM, Xing BS. 2019. The defense system for *Bidens pilosa* root exudate treatments in *Pteris multifida* gametophyte. Ecotox Environ Safe. 173:203–213. 10.1016/j.ecoenv.2019.01.097 30772710

